# Association of Food Security With Breastfeeding Practices: A Scoping Review

**DOI:** 10.7759/cureus.61177

**Published:** 2024-05-27

**Authors:** Christian H Guerrero, Rosa Cremades, Erick Sierra-Diaz, María de Lourdes López Flores, Lina María Murcia-Baquero, Elena Sandoval-Pinto

**Affiliations:** 1 Centro Universitario de Ciencias de la Salud, Departamento de Salud Pública, Universidad de Guadalajara, Guadalajara, MEX; 2 Centro Universitario de Ciencias de la Salud, Departamento de Microbiología y Patología, Universidad de Guadalajara, Guadalajara, MEX; 3 Centro Universitario de Ciencias Biológicas y Agropecuarias, Departamento de Biología Celular y Molecular, Universidad de Guadalajara, Guadalajara, MEX

**Keywords:** early initiation of breastfeeding, exclusive breastfeeding practice, food insecurity, "breastfeeding", food security

## Abstract

Breastfeeding is the fundamental, physiological, and psychosocial process by which the mother feeds the newborn. Early initiation of breastfeeding is recommended within the first hour of life and exclusive breastfeeding up to six months of age due to its optimal contribution of nutrients for the development of the newborn. Despite this, there are factors that affect this process which involve the nutritional, physical, and psychological state of the mother, such as food security or food insecurity, however, it is unknown if it will have a decisive impact on these factors concerning the cessation of breastfeeding or total duration of breastfeeding. This study is an in-depth review of the available information related to food security as a determinant in breastfeeding practices. We did a scoping review between December 2022 - January 2023. The principal inclusion criteria were: the use of the English language, qualitative and quantitative methods, and analytical studies. All the articles were available in full text and the manuscripts ranged from 1997 and 2022. Twelve studies were included: eight quantitative, two qualitative, and two mixed. In the quantitative studies, significant positive and negative associations were found between food insecurity, exclusive breastfeeding, early initiation of breastfeeding, cessation of breastfeeding, and total duration of breastfeeding. For their part, qualitative and mixed studies describe that women with severe food insecurity tend to feel weak and may have a poor perception of their diet and, consequently, their breastfeeding practices are lower. Moreover, there are qualitative studies that mention that the higher the food insecurity, the more frequently breastfeeding occurs. The inconsistency in the results may be due to factors involving the characteristics of each population, the instrument used to measure food security, and the variables by which the models were adjusted. It is necessary to carry out more studies on the subject since it is obvious that the relationship between the variables needs to be clarified.

## Introduction and background

The ideal feeding for newborns begins with breastfeeding, which is defined as a process in which physical, chemical, biochemical, hormonal and psychosocial exchange takes place, designed for the transfer of nutrients from the mother to the newborn, as well as the construction of a psychosocial bond between both [1]. There is scientific evidence about the advantages of breastfeeding for the newborn and the mother, and as well that it protects the newborn from diarrhea, gastroenteritis, and respiratory infections [2,3]. Likewise, a study explains its association with a reduction of 26%, 35% and 19%, with the likelihood of becoming overweight or obese, or developing type 2 diabetes and leukemia, respectively [2]. In addition, multiple studies have reported results indicating that breastfeeding reduces the risk of developing breast and ovarian cancer, type 2 diabetes, osteoporosis, and depression, and causes amenorrhea in the mother [3-5]. Breastfeeding contributes to the health and economy of the population through direct savings in the use of infant formulas and bottles, and indirectly contributes to a decrease in the prevalence of deaths in children and women, and in health care costs [6]. In addition, its consumption and production are environmentally friendly because it does not generate an ecological footprint [6]. The World Health Organization (WHO) recommends early initiation of breastfeeding, which consists of a supply of breast milk within the first hour after birth and exclusive breastfeeding during the first six months of life, which can be defined as only breast milk during this period, not including other liquids or foods, with a subsequent gradual introduction to food suitable for the infant for its age [1].

The United Nations International Children's Emergency Fund (UNICEF) reported that the prevalence of exclusive breastfeeding worldwide in 2008 was 35%, 2018 was 42% and 2022 it was 48% [7]. Conversely, the prevalence of early initiation of breastfeeding worldwide in 2005, 2017 and 2022 was 37%, 42% and 47%, respectively [7,8]. This shows that it has only increased around 10 percentage points in the last 15 years and they are less than half for both indicators of breastfeeding.

From the foregoing, we have a clear idea that early initiation of breastfeeding or breastfeeding in general can fail or not be carried out due to determining factors such as the influence of health professionals, the type of delivery (cesarean section or vaginal birth), lack of education about breastfeeding, educational and socioeconomic level, type of employment or need for rapid return to work, and food security [9-11]. Among these factors, food security stands out, which is defined as the accessibility to sufficient, safe, and good quality food so that all people may always satisfy their dietary needs and sustain a healthy and productive life. According to the Food and Agriculture Organization (FAO) in 2022, 2.4 billion people had moderate or severe food insecurity and 900 million were facing severe food insecurity. It is reported that more than 3.1 billion people cannot afford a healthy diet [12]. That being so, food insecurity could affect poor breastfeeding practices or insufficient milk production [9]. Food security is usually classified into four levels: food security, mild food insecurity, moderate food insecurity, and severe food insecurity [13]. Although food security is known to be a determinant of the quality of breastfeeding practices, information on the relationship is limited [9]. For this reason, it is essential to know the socioeconomic, cultural, educational, and, above all, nutritional conditions to guarantee successful breastfeeding [9,11]. Therefore, the aim of this study is to carry out an in-depth review of the available information related to food security as a determinant of breastfeeding practices.

## Review

Methods

Selection of Studies

This study used the methodology described by Arksey and O'Malley [14]. This has been the most commonly used method since 2005 and is widely cited in studies published in PubMed. First, a question was defined for the literature review, followed by an information search. PubMed was used as the main search engine. The keywords that were utilized were: food security, food insecurity, breastfeeding, and infant feeding practices. The number of articles resulting from the keyword combination was 629. Table 1 describes the number of matches and the results obtained.

**Table 1:** Search strategy and results obtained from PubMed.

	Keyword combination	Article result	Selected
PubMed	(breastfeeding) AND (food security)	218	9
PubMed	(breastfeeding) AND (food insecurity)	180	2
PubMed	(infant feeding practices) AND (food security)	231	1

Inclusion and Exclusion Criteria

The main reference selection criteria were: the use of the English language, qualitative and quantitative analytical studies, where at least one of the following variables was taken into consideration: early initiation of breastfeeding, exclusive breastfeeding, cessation of breastfeeding, and total duration of breastfeeding. All articles included were full texts. Reviews and manuscripts with only an abstract were excluded. The search, review, and selection of articles were carried out for two months (December 2022 - January 2023) and the manuscripts ranged between 1996 and 2022.

**Figure 1:**
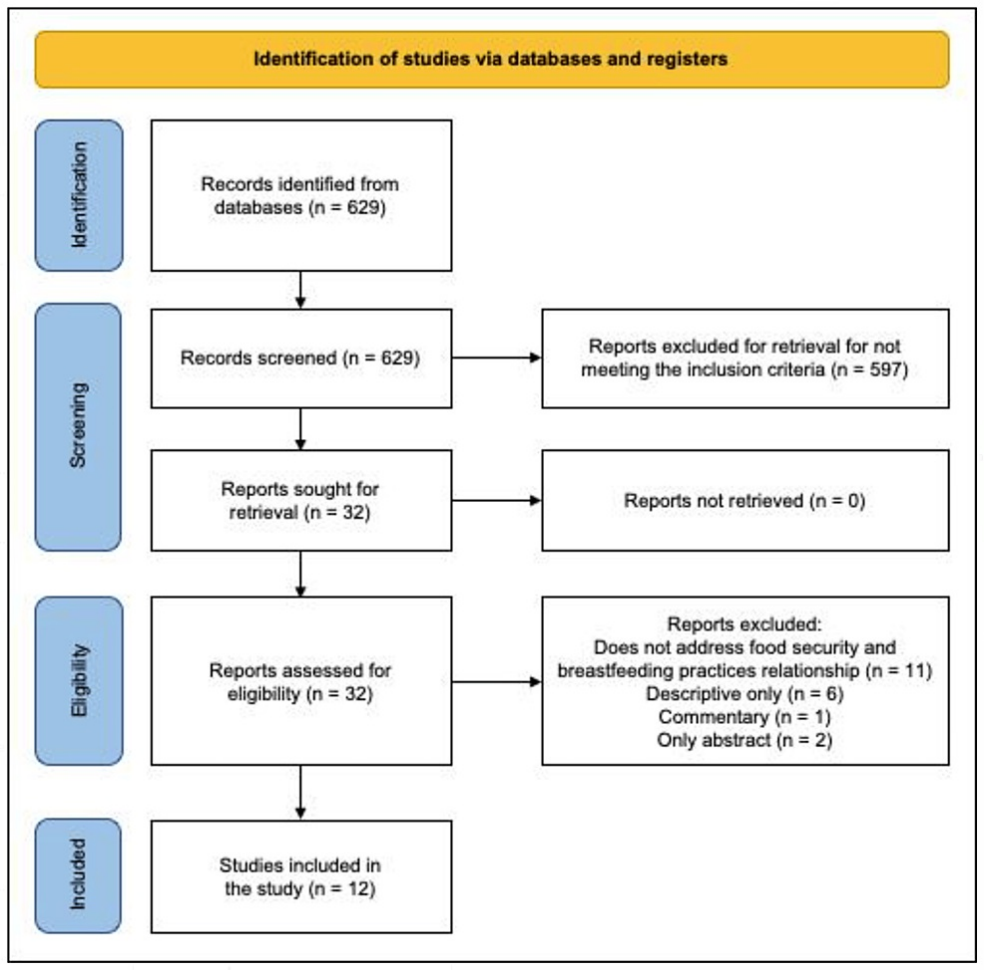
Preferred Reporting Items for Systematic Reviews and Meta-Analyses (PRISMA) flow diagram. [26]

Subsequently, 629 records were identified in the PubMed database, and based on the inclusion criteria, 597 were excluded for not meeting these criteria, of which 32 were taken to be evaluated and determine eligibility. Then 20 articles were discarded that did not talk about food security or breastfeeding, were only descriptive, comments, or had only the abstract. Therefore, the number of references included in the review was 12 (eight quantitative, two qualitative, and two quantitative and qualitative) (Table 2). This process is outlined in the Preferred Reporting Items for Systematic Reviews and Meta-Analyses (PRISMA) flow diagram (Figure 1) [26].

**Table 2:** Characteristics of the studies that were included.

Author (year) (ref.)	Country	Type of study	Sample	Study design
Miller et al. (2019) [15]	Kenya	Quantitative	122	Cohort
Orr et al. (2018) [16]	Canada	Quantitative	10450	Cohort
Dinour et al. (2020) [17]	USA	Quantitative	10159	Cross-sectional
Orozco et al. (2020) [18]	USA	Quantitative	2069	Cross-sectional
Wong et al. (2019) [19]	Canada	Quantitative	3838	Cross-sectional
Ezzeddin et al. (2019) [13]	Iran	Quantitative	325	Cross-sectional
Macharia et al. (2018) [20]	Kenya	Quantitative	1500	Cross-sectional
McIsaac et al. (2015) [21]	Canada	Quantitative	215	Cross-sectional
Sim et al. (2020) [22]	Canada	Qualitative	6	Cross-sectional
Gross et al. (2019) [23]	United States of America	Qualitative	100	Cross-sectional
Lesorogol et al. (2018) [24]	Haiti	Quantitative and qualitative	589	Cross-sectional
Webb-Girard et al. (2012) [25]	Kenya	Quantitative and qualitative	148	Cross-sectional

The selected manuscripts were analytically systematized into a matrix of extracts to easily organize them. For each article, a matrix of extracts was made that contained the following sections: main author, country of origin, date, design, sample characteristics, data analysis, and main results. Once all the articles were processed, a summary of the results was produced. After being divided into topics, the articles were systematized into an extract matrix that included: the lead author, year of publication, instrument used, and sociodemographic characteristics (Supplementary Table 1). Additionally, we created another table with the following information: associations in the adjusted final model for the quantitative interpretation or the summary of qualitative interpretation, the use of adjusted and operationalized variables in the final model, food security or insecurity, as well as breastfeeding practices (Supplementary Table 2). 

Results

The results of the analyzed articles were categorized into exclusive breastfeeding, early initiation of breastfeeding, and cessation of breastfeeding or total duration of breastfeeding. Subsequently, they were subcategorized by quantitative and qualitative studies.

Exclusive Breastfeeding

Quantitative studies: The WHO emphasizes that exclusive breastfeeding is the most desirable form of infant feeding in the first six months [1]. Since there is a relationship between food security, exclusive breastfeeding, and optimal health, Orr et al. (2018) found that among women who initiated breastfeeding, breastfeeding for up to six months differed markedly in household food insecurity status. In the adjusted model, early cessation of exclusive breastfeeding appeared to be negatively associated with moderate food insecurity (Hazard Ratios [HR] 1.24; 95% Confidence Interval [CI] 1.05-1.46) [16]. Similarly, Macharia et al. (2018) reported in the adjusted model that infants living in households were 104% (Odds Ratios [OR] 2.04; 95% CI 1.13-3.71) more likely to be exclusively breastfed up to six months of age compared with infants from households suffering from food insecurity [20]. Likewise, Webb-Girard et al. (2012) reported in adjusted multivariate models that women living with moderate or severe food insecurity were 2.7 times more likely to believe that they needed nutritionally adequate foods to produce breast milk that would help them maintain exclusive breastfeeding up to six months compared to women with food security or mild food insecurity (OR 2.7; 95% CI 1.0-7.3) [25].

Conversely, Ezzeddin et al. (2019) observed in the unadjusted model that women with food insecurity were 2.20 times more likely to exclusively breastfeed compared to women with food security (OR 2.20; 95% CI 1.35-3.57), however, this association was not significant in the final model (OR 1.41; 95% CI 0.74-2.69) [13]. Notwithstanding, Miller et al. (2019) reported that there is no association between severe food insecurity as a predictor of exclusive breastfeeding at six and 24 weeks postpartum, with a bivariate association of 0.47 (80% CI 0.20-1.10) and 1.55 (80% CI 0.52-4.61), respectively [15].

Qualitative studies: As in the previous section, the relationship between exclusive breastfeeding and food insecurity in qualitative studies was also analyzed. Gross et al. (2019) reported that mothers with food insecurity might avoid breastfeeding due to concerns about their unhealthy diets [23]. By contrast, Sim et al. (2020) note that their study participants described how they tried to follow expert guidance regarding exclusive breastfeeding, however, the lack of weight gain in their newborns motivated them to stop early [22]. Furthermore, Lesorolog et al. (2018) detailed that extreme food insecurity could lead to an increase in exclusive breastfeeding among mothers since they have the perception that it is their only resource to feed their neonates [24]. In contrast, the qualitative analysis conducted by Webb-Girard et al. (2012) mentions that there is a link between the lived experience of food insecurity related to hunger and the lack of confidence in successful exclusive breastfeeding [25].

Early Initiation of Breastfeeding

Quantitative studies: As in exclusive breastfeeding, food insecurity could have an impact on the initiation of breastfeeding. Thus, Dinour et al. (2020) found in the unadjusted model that women who were food insecure in the 12 months prior to delivery were less likely to have a total duration of breastfeeding compared with women who had food security (OR 0.67; 95% CI 0.54-0.82). However, this effect disappeared in the final model (OR 1.17; 95% CI 0.92-1.48) [17]. Additionally, Orozco et al. (2020) reported in the final model a non-significant increase in the odds of non-total duration of breastfeeding among households that experienced food insecurity in non-Hispanic Caucasians (OR 1.45; 95% CI 0.83-2.54), Hispanics (OR 1.10; 95% CI 0.55- 2.20) and non-Hispanic blacks (OR 0.82; 95% CI 0.50-1.33) [18].

Qualitative studies: Regarding the findings in qualitative studies, Gross et al. (2019) found that stress that occurs in women who present food insecurity is related to economic problems, and could therefore play an important role in the initiation of breastfeeding [23], while Sim et al. (2020) explained that despite their beliefs about the importance of breastfeeding and food security levels of the participants, each initiated the practice after delivery [22].

Cessation and Total Duration of Breastfeeding

Quantitative studies: In addition to the effect that food insecurity has on the initiation of breastfeeding, it also serves as a factor that could determine the cessation of breastfeeding or its total duration. Consequently, Wong et al. (2019) demonstrated in the unadjusted analysis a significant association between the total duration of breastfeeding and food insecurity (OR 0.98; 95% CI, 0.97-1.00). However, in the adjusted analysis they found no significant association [19]. At the same time, McIsaac et al. (2015) found a non-significant negative association between cessation of breastfeeding and food insecurity in the final model (HR 0.84; 95% CI 0.63-1.11) [21]. For his part, Dinour et al. (2020) found that women with food insecurity during a 12-month period had a 35% risk of stopping breastfeeding during the fourth and sixth week compared to women with food security (RR 0.65; 95% CI 0.50-0.85) [17].

Qualitative studies: Gross et al. (2019) argue that women who have experienced stressful events resulting from food insecurity, as related to economic factors, present a decrease in the duration of breastfeeding, coupled with an erroneous perception of its effect on the quality of breastfeeding [23]. In the same manner, Lesorolog et al. (2018) found that a mother with food insecurity who could not afford enough food for herself and her infant decided to discontinue breastfeeding due to weakness and the perception of insufficient breast milk production [24].

Discussion

Breastfeeding is a natural physiological process that represents the completion of the reproductive cycle of women [27]. Moreover, it is an essential and fundamental human right for the optimal growth of a human being that is affected from birth to adulthood [19]. As established by the United Nations Organization (UNO) and their convention on the rights of the child, every infant and child has the right to good nutrition including breastfeeding [28]. In the words of the WHO and their systematic review, it is recommended that an optimal duration of exclusive breastfeeding be for at least six months, and early initiation of breastfeeding within the first hour of being born, in order to rule out any adverse effects on growth in babies and guarantee the benefits to their health [29]. In this study, we analyzed the most relevant information regarding food security or insecurity and its relationship with breastfeeding practices. Hence, we found that this association exists, as was reported by multiple authors [16,17,20,22-25]. Moreover, Orr et al. (2018) found an association between the initiation and duration of breastfeeding and their level of food security [16].

However, some factors contribute to early initiation of breastfeeding and exclusive breastfeeding which are unsuccessful and of shorter duration [9]. According to scientific evidence, there are multiple factors associated with the initiation, continuation, and cessation of breastfeeding such as being overweight and obese, education, employment status and income, ethnicity, depression, anxiety, smoking, support, mode of delivery, maternal breastfeeding education, dyad separation, and parity [30,31].

Among these factors is food security, which exists when all people have, at all times, physical, social, and economic access to sufficient, safe, and nutritious food that meets their daily energy needs and food preferences, to lead an active and healthy life [32]. Therefore, the purpose of this study was to review the published scientific evidence related to these variables.

The results of exclusive breastfeeding indicate that it could be associated with positive food insecurity or have a negative impact on food security. Gross et al. (2019), Orr et al. (2018), and Webb-Girard et al. (2012) explain that a high level of food insecurity increases the risk of stopping exclusive breastfeeding since these women have the idea that their level of food insecurity leads them to not have an adequate nutritional status to carry out successful exclusive breastfeeding for the first six months of life [16,23,25]. The aforementioned agrees with Macharia et al. (2018), who reported that households living with food security were more likely to have exclusive breastfeeding up to six months of age [20]. Inversely, Ezzeddin et al. (2019) found that introducing food insecurity could promote exclusive breastfeeding, although not significantly [13]. This may be strengthened by the findings of Lesorolog et al. (2019), where it is mentioned that mothers with food insecurity may have a perception that exclusive breastfeeding is a valuable resource for maintaining adequate health for their children [24]. Conversely, the study by Miller et al. (2019) mentions that food insecurity is not a predictor of exclusive breastfeeding [15].

Regarding the early initiation of breastfeeding, Dinour et al. (2020) found that women with food insecurity during the 12 months leading up to childbirth have non-significant lower probabilities of initiating breastfeeding, and suggest that this is because food insecurity can lead to maternal weakness and a perceived amount of insufficient milk, related to an inadequate nutritional status and due to a poor maternal diet, similar to what Gross et al. describes (2019) [17,23]. Furthermore, these results are similar to those of a study done by Orozco et al. (2020) in which food insecurity could affect the early initiation of breastfeeding regardless of the ethnicity of the mother [18]. Conversely, the mothers in the qualitative study by Sim et al. (2020) started breastfeeding after delivery, regardless of the beliefs they may have had about this practice [22]. 

Lastly, Dinour et al. (2020) show that women with prenatal food insecurity have higher risks of stopping breastfeeding. This substantiates the findings of Gross et al. (2019) and Lesorolog et al. (2018), which explain that this may be due to the perception of the relationship between an unhealthy diet and the quality of breast milk [17,23,24]. In contrast, Wong et al. (2019) and McIsaac et al. (2015) found that mothers with food insecurity could be less likely to stop breastfeeding [19,21].

As we mentioned previously, there are multiple factors related to breastfeeding practices. Although in this review we have focused on food security, some reports show the main social, physical, and mental factors which, together with food security, must be taken into consideration to understand complex relationships, their association, and how they are influencing this process [30].

To our knowledge, there is no review that analyzes the scientific evidence of quantitative studies as to their effects on food insecurity, or the main indicators of breastfeeding. Nonetheless, the review was instrumental in pointing out possible gaps that still exist in the research on this topic. Thus, the present study provides valuable information on the impact generated by food insecurity regarding exclusive breastfeeding, early initiation of breastfeeding, cessation of breastfeeding, and the ideal duration of breastfeeding. Therefore, these results should be considered by health professionals and public health policy administrators, to contribute to the improvement of social programs that allow successful breastfeeding. Additionally, the recommended method of Arksey and O'Malley was used for the literature review [14]. However, within the limitations of the study, there is the loss of some publications that were not identified in the search because their published language was not in English.

## Conclusions

It can be concluded that the published evidence confirms to a greater or lesser extent the relationship between breastfeeding and the level of food security or food insecurity that the mother presents before, during, and after delivery. In general, several authors found that food insecurity is related to misinformation regarding breastfeeding, which contributes to a poor perception of it and, therefore, women with unhealthy eating habits prefer to stop or reduce the duration of breastfeeding. However, the results are inconsistent due to several specific factors that occur in each population, in addition to the different instruments that were used to measure food security and the characteristics that differ at the time of analysis of the quantitative studies. 

Due to the complex relationship that may exist between breastfeeding and food security, it is recommended that more epidemiological studies be carried out that contribute to the generation of scientific evidence that can aid in learning more about the effects of food security in relation to breastfeeding practices. It would therefore be necessary to implement programs that allow for the timely detection of the mother's food insecurity status in order to make the necessary modifications, thus reducing the risk of affecting breastfeeding practices and a human need for being a declared right by the UNO.

## Appendices

**Table 3:** Supplementary Table. Instrument and sociodemographic characteristics associations NA: No available. SD: Standard deviation. Q1: First quarter’ Q3: Third quarter

Author (year) (ref.)	Instrument used to measure food security	Age of mothers	Marital status	Income	Education	Employment	Age of children	Biological sex of children
Miller et al. (2019) [15]	Household food insecurity access scale (HFIAS) for measurement of food access by the U.S. Agency for International Development (https://pdf.usaid.gov/pdf_docs/Pnadk896.pdf)	Mean (SD) 25.2 (6.2)	Single: 22.24	NA	NA	Mean (SD) 6 weeks (8)	Female: 58.025
			Married: 96.76				>Secondary: 26.968	Male: 101.975
Orr et al. (2018) [16]	Household food Security survey module and health Canada’s coding method (www.cmaj.ca/lookup/suppl/doi:10.1503/cmaj.170880/-/DC1)	Mean (SD) 30.8 (0.2)	Married or common-law: 9279.6	Mean (SD) 44050 (966)	Postsecondary graduation: 7471.75	NA	NA	NA
			Single, divorced, separated, widowed: 2978.25		Less than postsecondary graduation: 2978.25			
Dinour et al. (2020) [17]	Household food security by the U.S. Department of Agriculture, Economic Research Service, 2017 version (https://www.ers.usda.gov/publications/pub-details/?pubid=90022)	20-24: 2279	Not married: 3372	$0-2200: 3390	Years	NA	10 weeks-4 months old	NA
		25-29: 3158		$22001-37000: 1685	0-11: 971			
				$37001-52000: 1178	12: 2261			
		30-34: 3021	Married: 6787	$52001-67000: 900	13-15: 3105			
		35+: 1701		$67001+: 3006	16+: 3822			
Orozco et al. (2020) [18]	Guide to measuring household food security by the U.S. Department of Agriculture, 2000 version (https://nhis.ipums.org/nhis/resources/FSGuide.pdf)	Mean (SD) 27.5 (6.2)	NA	>130%: 943	College or more: 434	NA	Months	Female: 1043
					Some college: 585		0-5.9: 621	
				≤130%: 948	High school: 485		6-11.9: 595	Male: 1026
					Less than high school: 516		12-24: 853	
Wong et al. (2019) [19]	1-item food insecurity screen from NutriSTEP questionnaire (Randall Simpson JA, Keller HH, Rysdale LA, Beyers JE. Nutrition Screening Tool for Every Preschooler (NutriSTEP): validation and test-retest reliability of a parent-administered questionnaire assessing nutrition risk of preschoolers. Eur J Clin Nutr. 2008 Jun;62(6):770-80. doi: 10.1038/sj.ejcn.1602780. Epub 2007 Jun 6. PMID: 17554250) and a 2-item screen from 18-item Household Food Security Survey in the U.S., 2010 version (https://www.ers.usda.gov/webdocs/publications/44906/6893_err125_2_.pdf?v=0)	Mean (SD) 34.8 (4.45)	Single parent family: 131	$0-29999: 164	Primary school: 36	No employment: 726	Mean (SD) 23.2 months old (9.7)	Female: 1838 Male: 2000
				$30000-79999: 489	High school: 280	Full-time employed: 1451		
			No single parent family: 3568	$80000-150000: 990	College: 3431	Parental leave: 932		
				Over $150000: 1342		Part-time employed: 425		
						Self-employed: 53		
Ezzeddin et al. (2019) [13]	Assessing the internal validity of a household survey-based food security measure adapted for use in Iran (Rafiei M, Nord M, Sadeghizadeh A, Entezari MH. Assessing the internal validity of a household survey-based food security measure adapted for use in Iran. Nutr J. 2009 Jun 26;8:28. doi: 10.1186/1475-2891-8-28. PMID: 19558676; PMCID: PMC2714524.)	Mean (SD) 28.6 (5.6)	NA	NA	Under high school diploma: 71	Employment: 44	Months	Female: 169
							3: 40	
					High school diploma or higher: 254	Housekeepers: 281	3-6: 202	Male: 154
							6-8: 83	
Macharia et al. (2018) [20]	Household food insecurity access score method (does not specify nor the reference)	14-20: 312.68	Not in a union: 179.46	Poorest: 271.947	Less than primary: 197.079	Not working: 765.195	6-11 months old	Female: 524.076
		21-24: 339.108	In a union: 921.53	Middle: 226.806	Primary school: 606.651	Working: 300.573		Male: 561.51
		25-29: 267.543		Least poor: 252.129	Secondary school: 262.038			
		30-45: 166.251:	Missing: 1.10	Missing: 350.118	Missing: 35.232	Missing: 36.66		Missing: 16.515
		Missing: 15.414						
McIsaac et al. (2015) [21]	Food security module (modified version) by the U.S. Department of Agriculture, 2000 version (https://nhis.ipums.org/nhis/resources/FSGuide.pdf)	NA	NA	Receives income support: 96	NA	NA	Mean (SD) 4 years old (0.8)	Female: 115
				Not receives income support: 119				Male: 100
Sim et al. (2020) [22]	Semi-structured interview guide (Gubrium, Jaber F., and James A. Holstein. Postmodern Interviewing Thousand Oaks, CA: SAGE Publications, Inc.; 2003. doi:10.4135/9781412985437)	18 or older	Single: 3	NA	NA	NA	NA	NA
Gross et al. (2019) [23]	Core food security module by the U.S. Department of Agriculture, 2000 version (https://nhis.ipums.org/nhis/resources/FSGuide.pdf)	Mean (SD) 30 (6)	Married or living as married: 76	Difficulty paying bills: 20	Educational attainment (less than high school): 41	NA	3-24 months old	NA
Lesorogol et al. (2018) [24]	Semi‐structured interviews guide by a qualitative research specialist (not referenced)	NA	NA	Mean (SD) Income money per day: 3.48 (3.09)	NA	Any employed: 303.335	6-11 months old	NA
						Market or small business: 211.451		
Webb-Girard et al. (2012) [25]	Household food insecurity access scale (HFIAS) for measurement of food access by the U.S. Agency for International Development (https://pdf.usaid.gov/pdf_docs/Pnadk896.pdf)	Median (Q1, Q3) 26 (21, 30)	NA	Kenyan shilling: 4200 (2000-7000)	Schooling year median (Q1, Q3): 8 (7, 12)	NA	<6 months old: 25.22	NA

**Table 4:** Supplementary Table. adjustment variables and operationalization of the variables NA: No available. CI: Confidence interval.

Author (year) (ref.)	Adjusted models or interpretation							Adjusted variables	Operationalized of food security/insecurity		Operationalized of breastfeeding variables	
Miller et al. (2019) [15]	Bivariate associations (80% CI) household food insecurity			Predictors of exclusive breastfeeding at 6 weeks postpartum		Predictors of exclusive breastfeeding at 24 weeks postpartum		NA	Categorical	Household food insecurity moderate	Categorical	Exclusive breastfeeding for 6 and 24 weeks yes/no
				Moderate: (reference)		Moderate: 1.55 (0.70-3.44), p value: 0.337				Household food insecurity high		
				High: 0.47 (0.20-1.10), p value: 0.256		High: 1.55 (0.52-4.61), p value: 0.238						
Orr et al. (2018) [16]	Risk of early cessation (<6 months) of exclusive breastfeeding household food insecurity status hazard ratios (95% CI)			Secure: (reference)	Odds ratios exclusively breastfed to ≥6 months (95% CI)	Food security: (reference)		Age, education, partnership status, immigrant status, number of children < 18 years of age, income (adjusted for household size), Aboriginal identity and survey year, presence of mood disorder, presence of diabetes mellitus.	Categorical	Food security	Categorical	Breastfeeding initiation yes/no
				Marginally insecure: 1.17 (1.05-1.46)		Marginal food insecurity: 0.80 (0.52-1.22)				Marginal food insecurity		
				Moderately insecure: 1.24 (1.05-1.46)		Moderate food insecurity: 0.61 (0.40-0.93)				Moderate food insecurity	Continuous	Duration of exclusive breastfeeding
				Severely insecure: 1.19 (0.96-1.49)		Severe food insecurity: 0.60 (0.30-1.20)				Severe food insecurity		
Dinour et al. (2020) [17]	Binomial regression predicting breastfeeding initiation odds ratios (95% CI)		Food secure: (reference)	Multinomial logistic regression using breastfeeding ≥10 weeks as reference (95% CI)		Food secure: (reference)		Prenatal food security status to assess the unadjusted relationship between the independent and dependent variables, socioeconomic status variables: maternal age, income, marital status, maternal education, maternal race/ethnicity, insurance type at the time of survey, breastfeeding information provided by a healthcare provider, and WIC status during pregnancy, postpartum depression, number of stresses during pregnancy, and pregnancy intention, delivery method, Kotelchuck index, length of hospital stay, and current smoking status.	Categorical	Food secure	Categorical	Breastfeeding initiation yes/no
						Food insecure (breastfeeding <1 week ): 0.92 (0.61-1.37)					Categorical	Early breastfeeding cessation/Breastfeeding duration
						Food insecure (breastfeeding 1-3 weeks): 0.93 (0.72-1.20)						< 1 week
												1-3 weeks
			Food insecure: 1.17 (0.92-1.48)			Food insecure (breastfeeding 4-6 weeks): 0.65 (0.50-0.85)				Food insecure		4-6 weeks
												7-9 weeks
						Food insecure (breastfeeding 7-9 weeks): 1.03 (0.79-1.34)						≥10 weeks
Orozco et al. (2020) [18]	Odds ratios for not initiating breastfeeding among food insecure households (95% CI)			Food secure: (reference)				Household food security status, infant’s sex (male vs female), household reference person’s education level (college or more vs some college vs high school vs less than high school), income-to-poverty ratio (>130% vs ≤130%), current participation in the Special Supplemental Nutrition Program for Women, Infants, and Children (yes vs no), and mother’s age at the time of delivery (continuous), account for acculturation, nativity status (born in the US vs born outside the US).	Categorical	Food secure	Categorical	Initiation of breastfeeding yes/no
				Non-Hispanic White: 1.45 (0.83-2.54), p value: 0.19								
				Hispanic: 1.10 (0.55-2.20), p value: 0.79						Food insecure		
				Non-Hispanic: 0.82 (0.50-1.33), p value: 0.41								
Wong et. al. (2019) [19]	Logistic regression model for total breastfeeding duration (per month) odds ratios (95% CI)			0.988 (0.950-1.028), p value: 0.558				Child age, child sex, maternal age, maternal ethnicity, maternal education, maternal employment, number of children, single-parent family, neighborhood equity score, self-reported income, interaction between total breastfeeding duration and family self-reported income.	Categorical	Food insecurity yes/no	Continuous	Total breastfeeding duration
Ezzeddin et al. (2019) [13]	Logistic regression for exclusive breastfeeding odds ratios (95% CI)			Food insecure: (reference)				NA	Categorical	Food secure	Categorical	Exclusive breastfeeding
				Food secure: 1.41 (0.74-2.69), p value: 0.292						Food insecure		Non-exclusive breastfeeding
Macharia et al. (2018) [20]	Logistic regression for relationship between exclusive breastfeeding at 6 months and household food security odds ratios (95% CI)			Food insecure: (reference)				Socioeconomic status, marital, mother’s age, religion, education, parity, ethnicity, health facility, birth weight.	Categorical	Food secure	Categorical	Exclusive breastfeeding up to 6 months
				Food secure: 2.04 (1.13-3.71), p value: 0.019						Food insecure		Exclusive breastfeeding < 6 months
McIsaac et al. (2015) [21]	Risk of breastfeeding cessation for food insecure relative to food secure households cox proportional hazards ratios (95% CI)			Food secure: (reference)				Probability of participant selection in each community, socio-economic position indicators: house in need of repairs, living in public housing, receiving income support; child health indicators: birth weight, caregiver-rated health, child body mass index centile; traditional knowledge indicators: hunter in household, spoken inuit language.	Categorical	Food secure	Continuous	Breastfeeding duration/cessation
				Food insecure: 0.84 (0.63-1.11)						Food insecure		
Sim et al. (2020) [22]	All participants discussed their desire to breastfeed through their belief that breastfeeding was the most natural and healthy way to feed their babies, while formula was presented as an unaffordable and less suitable alternative. In accordance with their beliefs about the importance of breastfeeding, each of the participants began the practice after giving birth. Breastfeeding was also consistently positioned as a taken-for-granted practice associated with an ideal maternal body. The experience of living with income-related food insecurity and high body weight also contributed to participants' construction of the ideal maternal physical body as well-nourished and of “normal” shape and size. Participants shared the perspective that maternal excess weight was a failure on both a personal and moral level, and this was implicated in the practice of breastfeeding.							NA	NA		NA	
Gross et al. (2019) [23]	Common concerns among mothers of young babies related to breastfeeding. Mothers may avoid breastfeeding due to concerns about their own diet. Mothers perceived their diet as poor and were concerned that if they did not eat enough fruits and vegetables, their breast milk would be of low quality and lack necessary nutrients. Mothers experienced substantial stress related to food insecurity and difficulty paying bills. Some mothers felt that stress would decrease the amount of breast milk or cause the milk to "dry up." Mothers were concerned that if breast milk was insufficient, formula would be needed, which was expensive and not always available.							NA	NA		NA	
Lesorogol et al. (2018) [24]	Women reported that unstable and low-paid employment and lack of access to social and economic support tend to have difficulty accessing sufficient food and other basic needs for themselves and their children, resulting in a situation of food insecurity. Food insecurity, in turn, influenced breastfeeding practices in different ways. Some mothers who could not afford enough food for themselves decided to stop breastfeeding due to weakness and perceived insufficiency of breast milk. Some mothers reported increased breastfeeding when they could not afford enough food for their children.							NA	NA		NA	
Webb-Girard et al. (2012) [25]	Quantitative Odds ratios (95% CI)	Women need adequate food to exclusive breastfeeding for 6 months: 2.7 (1.0-7.3)		Qualitative			There is a link between the lived experience of hunger-related AI and lack of confidence in successful exclusive breastfeeding.	Maternal age, parity, years of schooling, pregnancy status, respondent contributes to household income, household size and whether the mother received infant feeding counseling.	Categorical	Food secure/mildy food insecure	Categorical	Exclusive breastfeeding for 6 months yes/no
										Moderate/severely food insecure		
